# Supplementary figures and images for: Defective mesenchymal Bmpr1a-mediated BMP signaling causes congenital pulmonary cysts
Source: eLife. 2024 Jun 10;12:RP91876. doi: 10.7554/eLife.91876 (PMC11164533; doi:10.7554/eLife.91876)

B

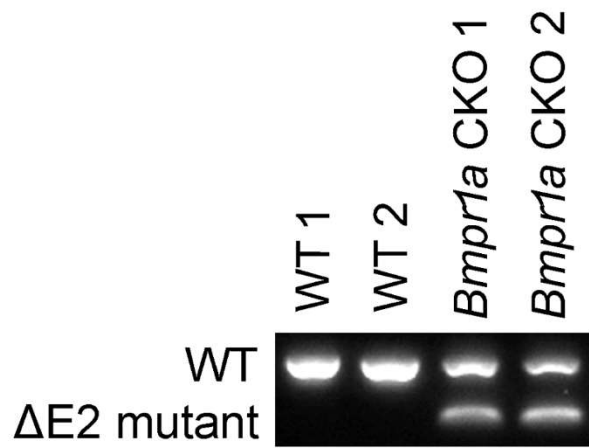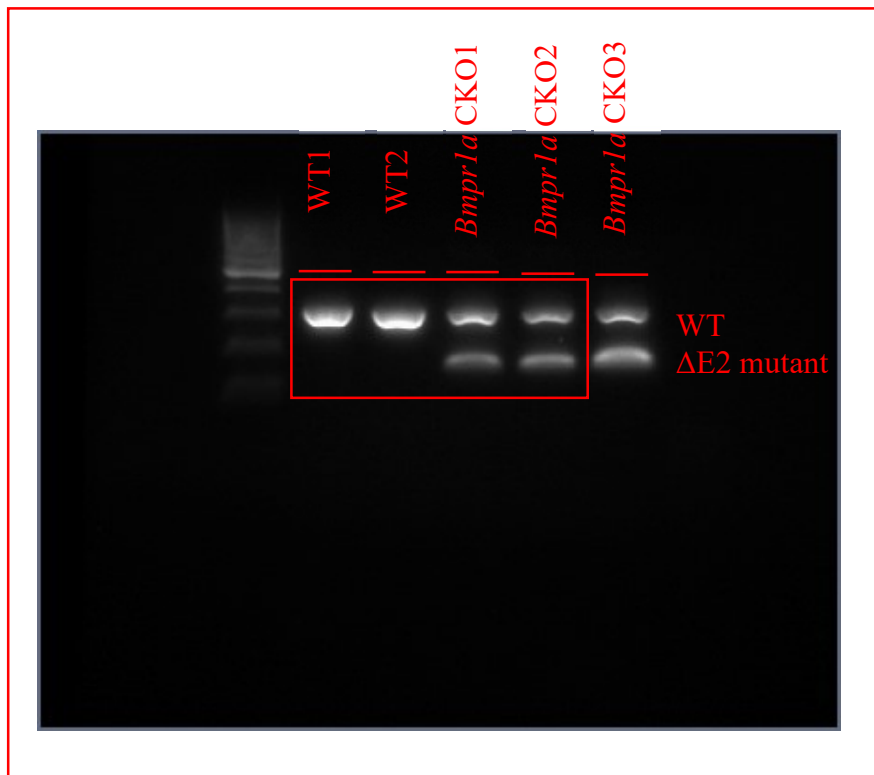

Figure 1-figure supplement 1

Supplement: Figure 1—figure supplement 1—source data 2. [file elife-91876-fig1-figsupp1-data2.zip › Figure 1-figure supplement 1-source data 2.pdf]

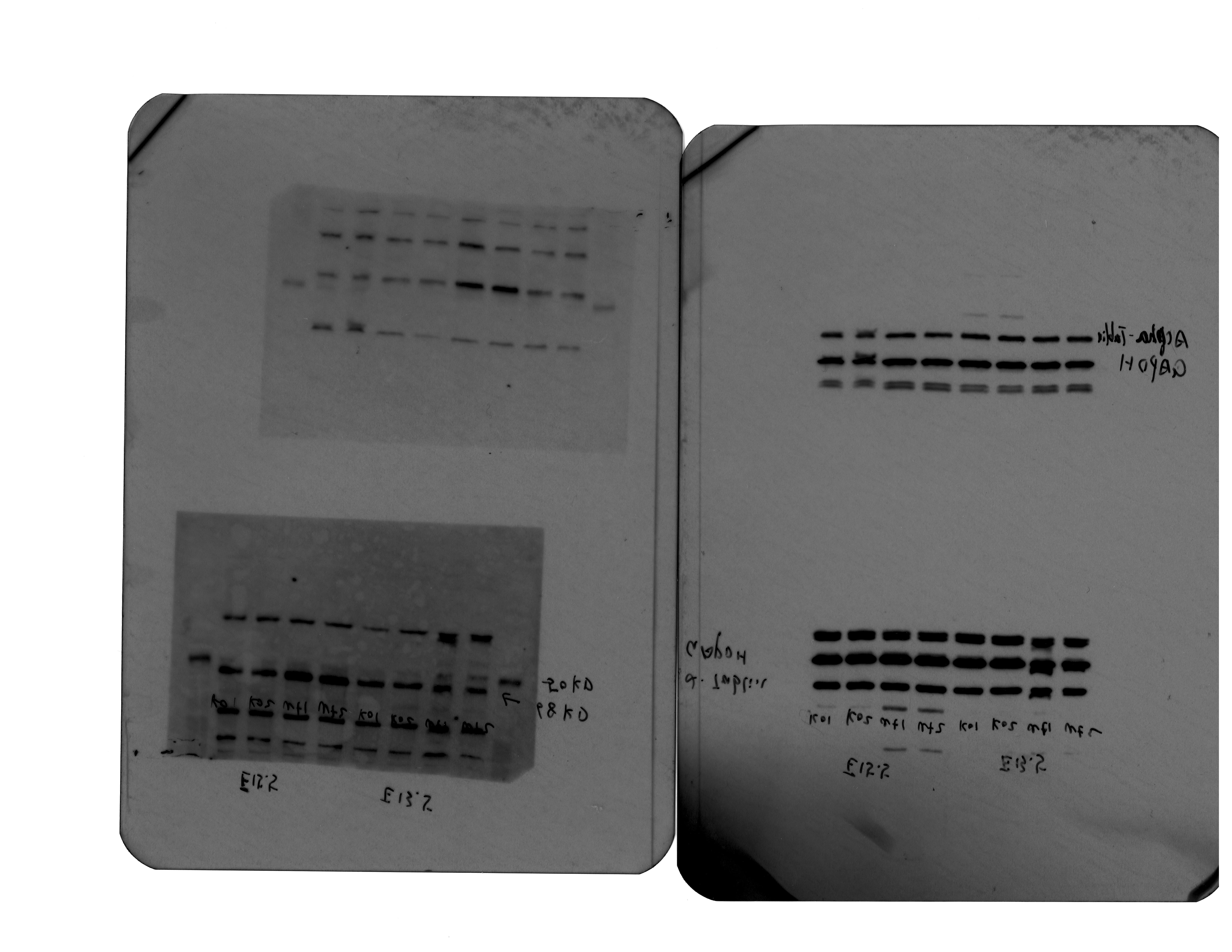

Supplement: Figure 3—source data 1. [file elife-91876-fig3-data1.zip › Figure 3-source data 1.tif]

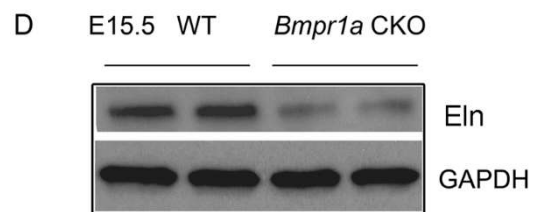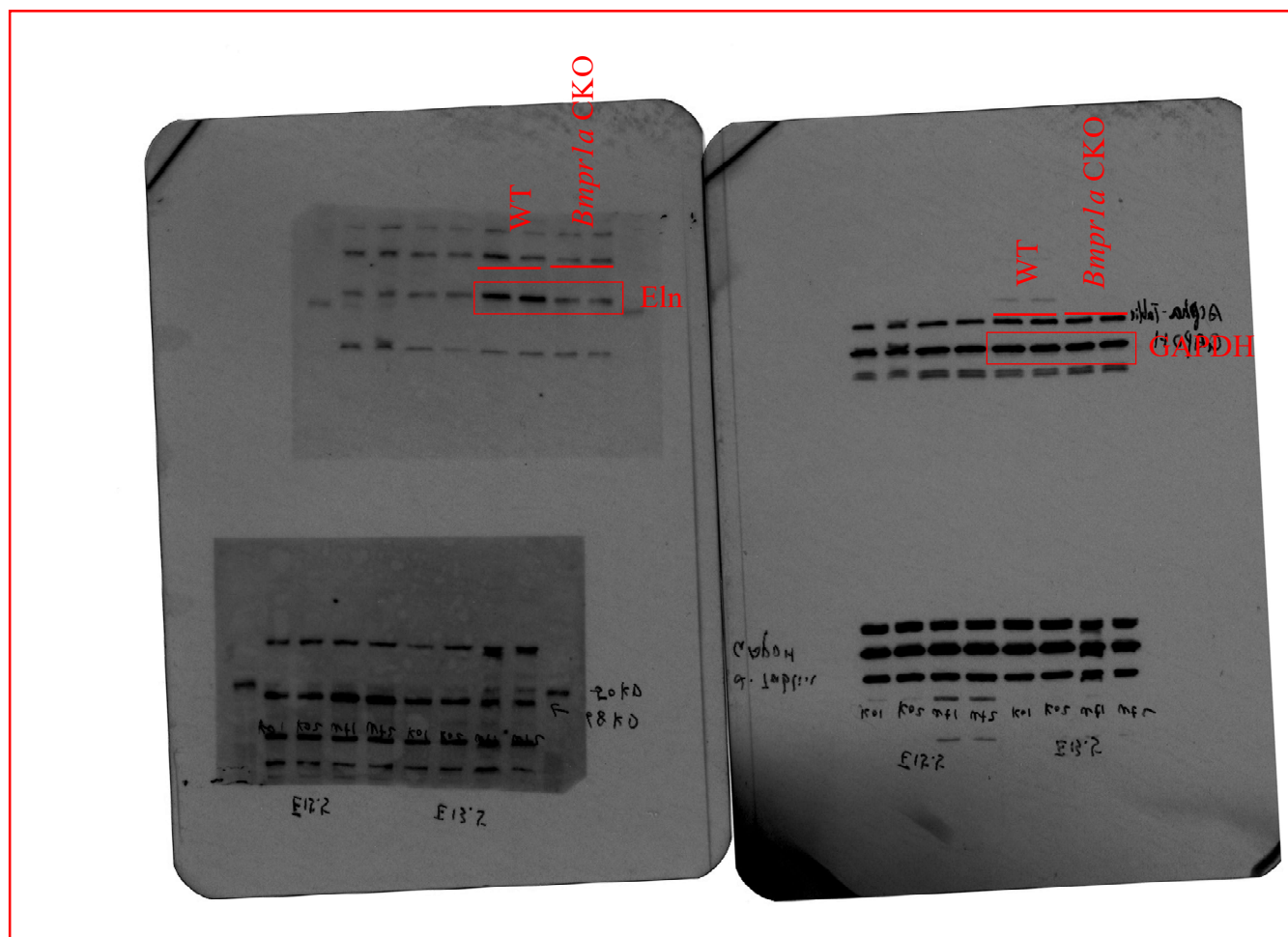

Figure 3

Supplement: Figure 3—source data 2. [file elife-91876-fig3-data2.zip › Figure 3-source data 2.pdf]

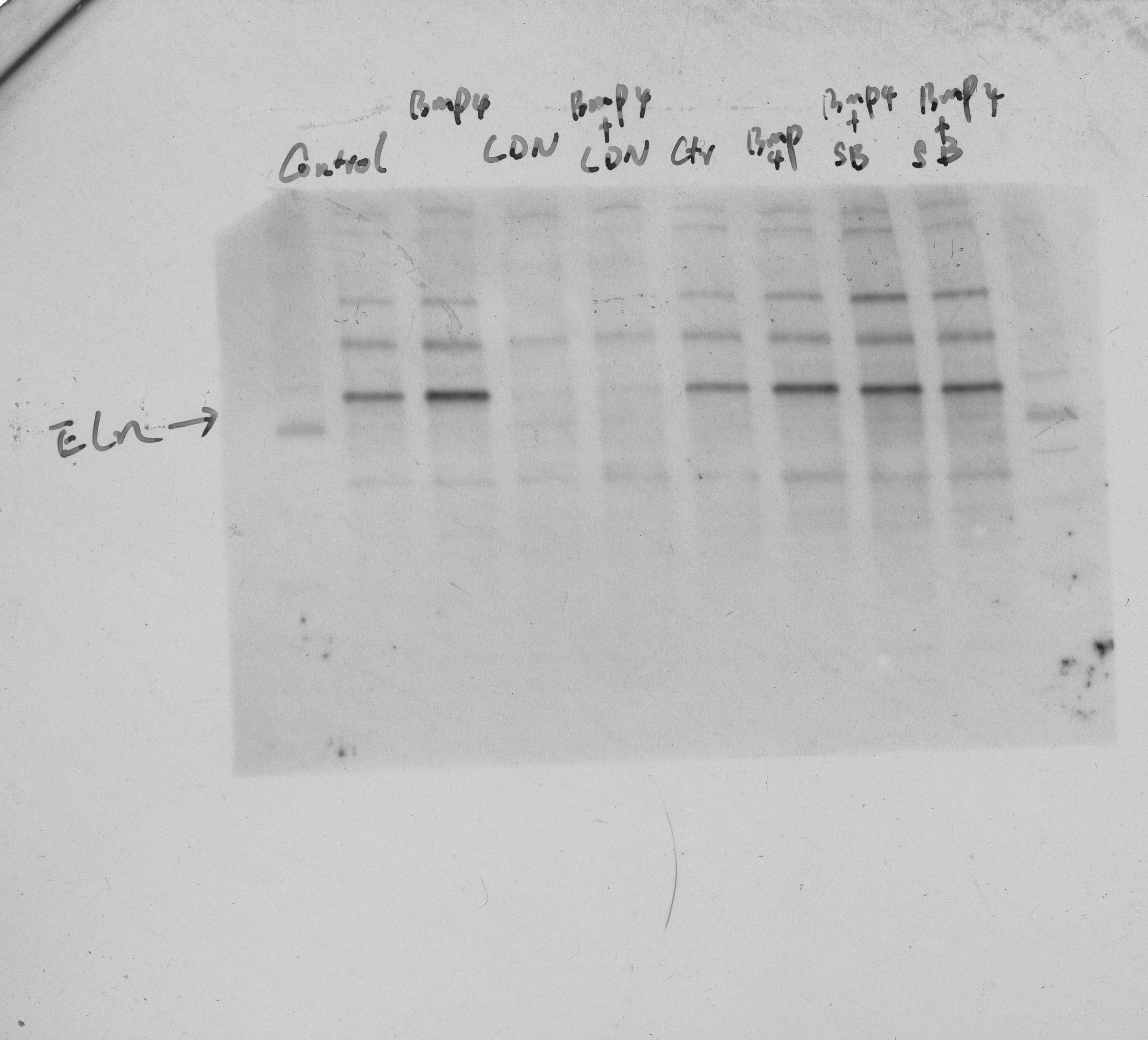

Supplement: Figure 3—source data 3. [file elife-91876-fig3-data3.zip › Figure 3-source data 3.tif]

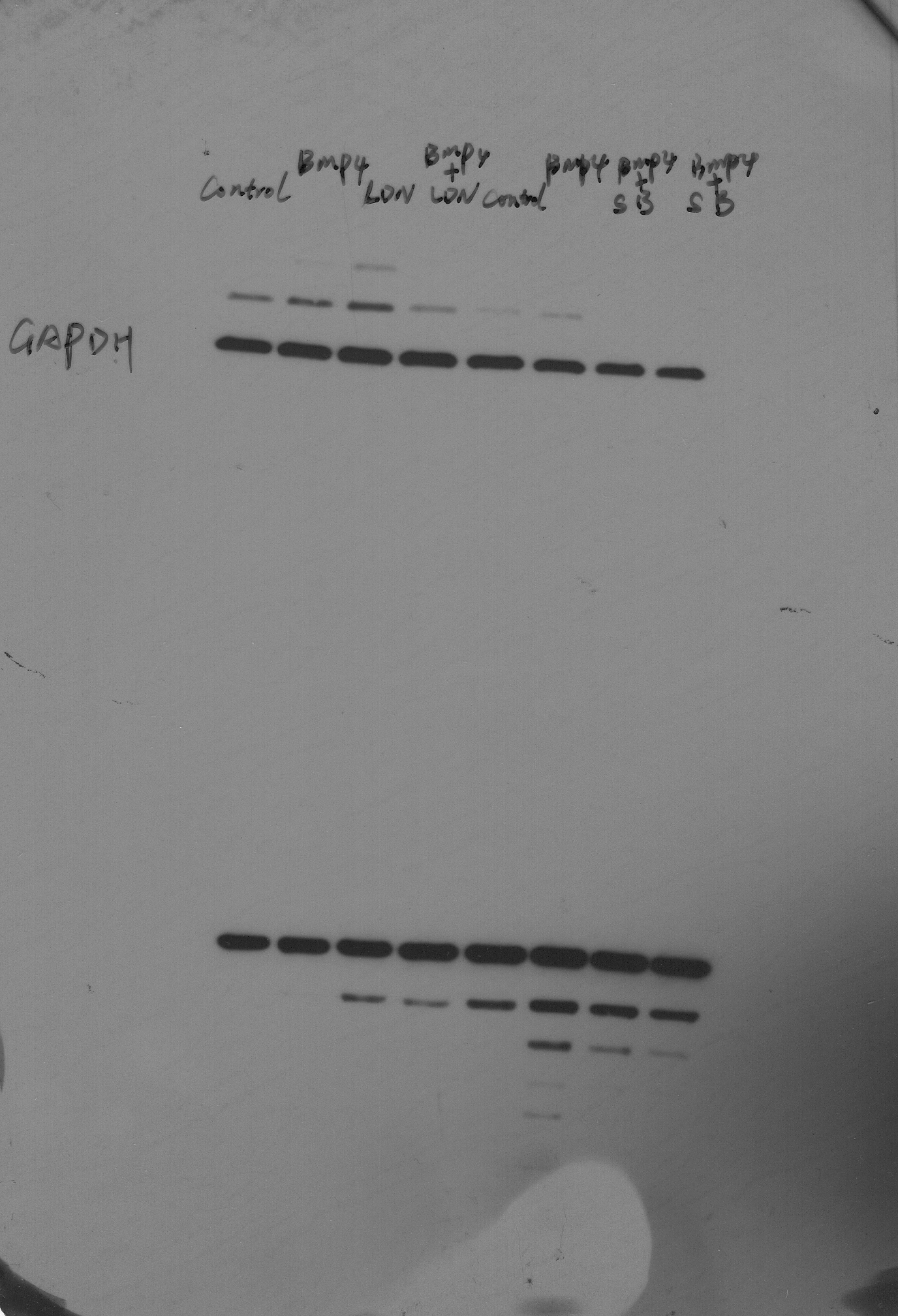

Supplement: Figure 3—source data 4. [file elife-91876-fig3-data4.zip › Figure 3-source data 4.tif]

E

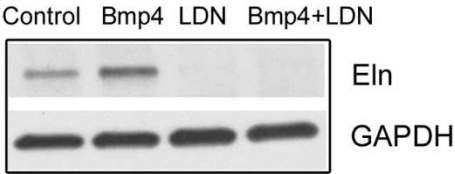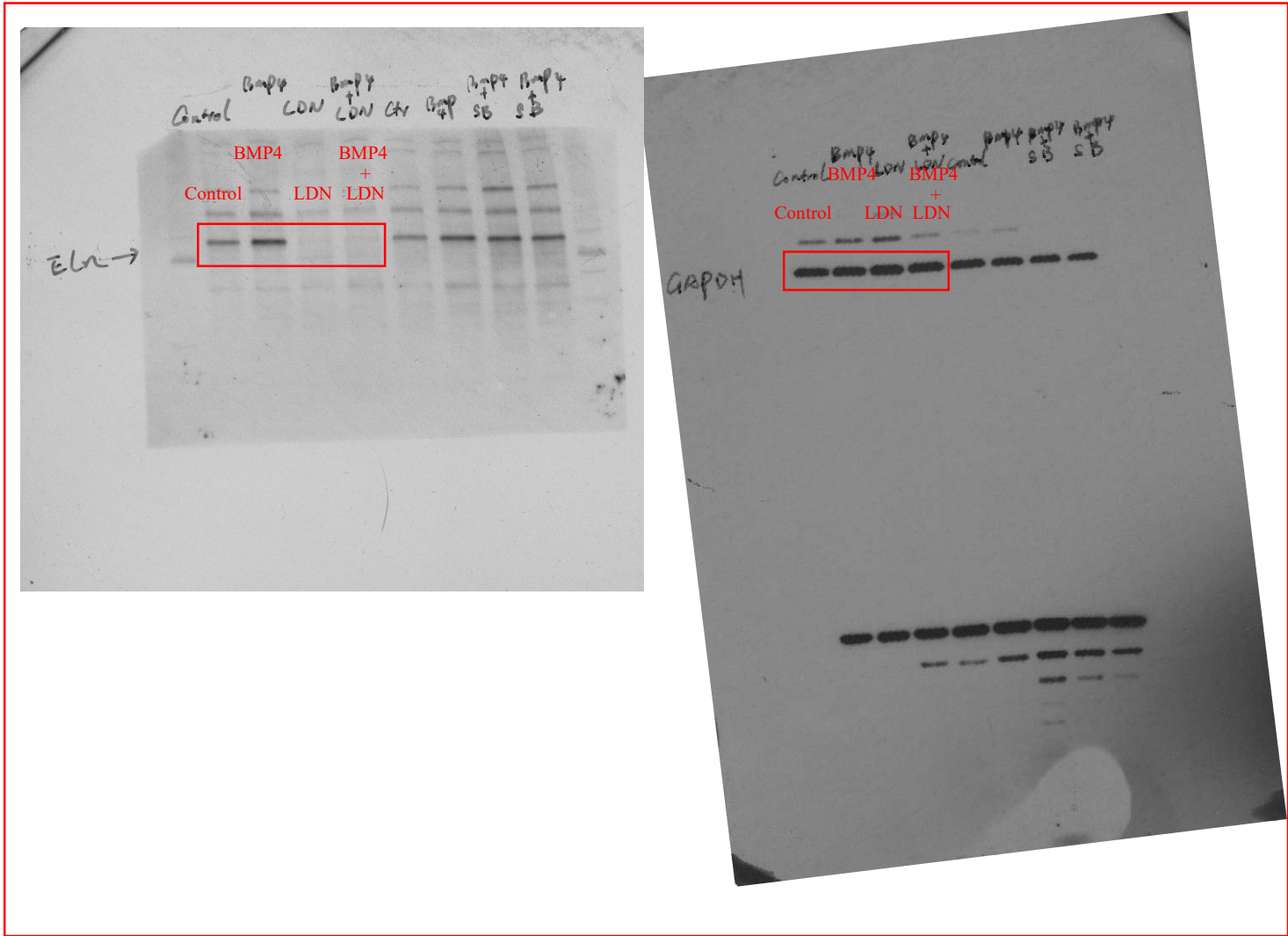

Figure 3

Supplement: Figure 3—source data 5. [file elife-91876-fig3-data5.zip › Figure 3-source data 5.pdf]

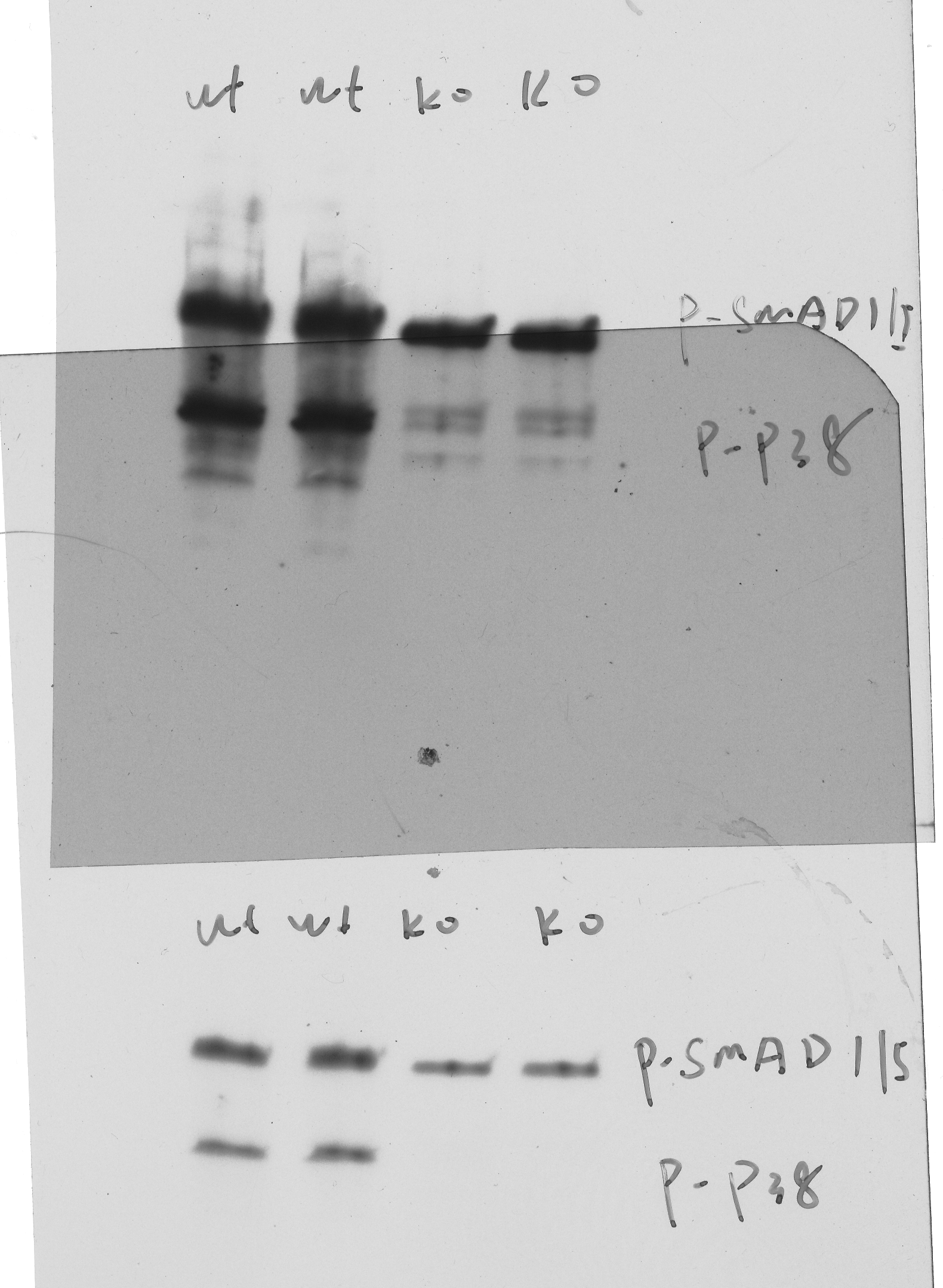

Supplement: Figure 4—source data 1. [file elife-91876-fig4-data1.zip › Figure 4-source data 1.tif]

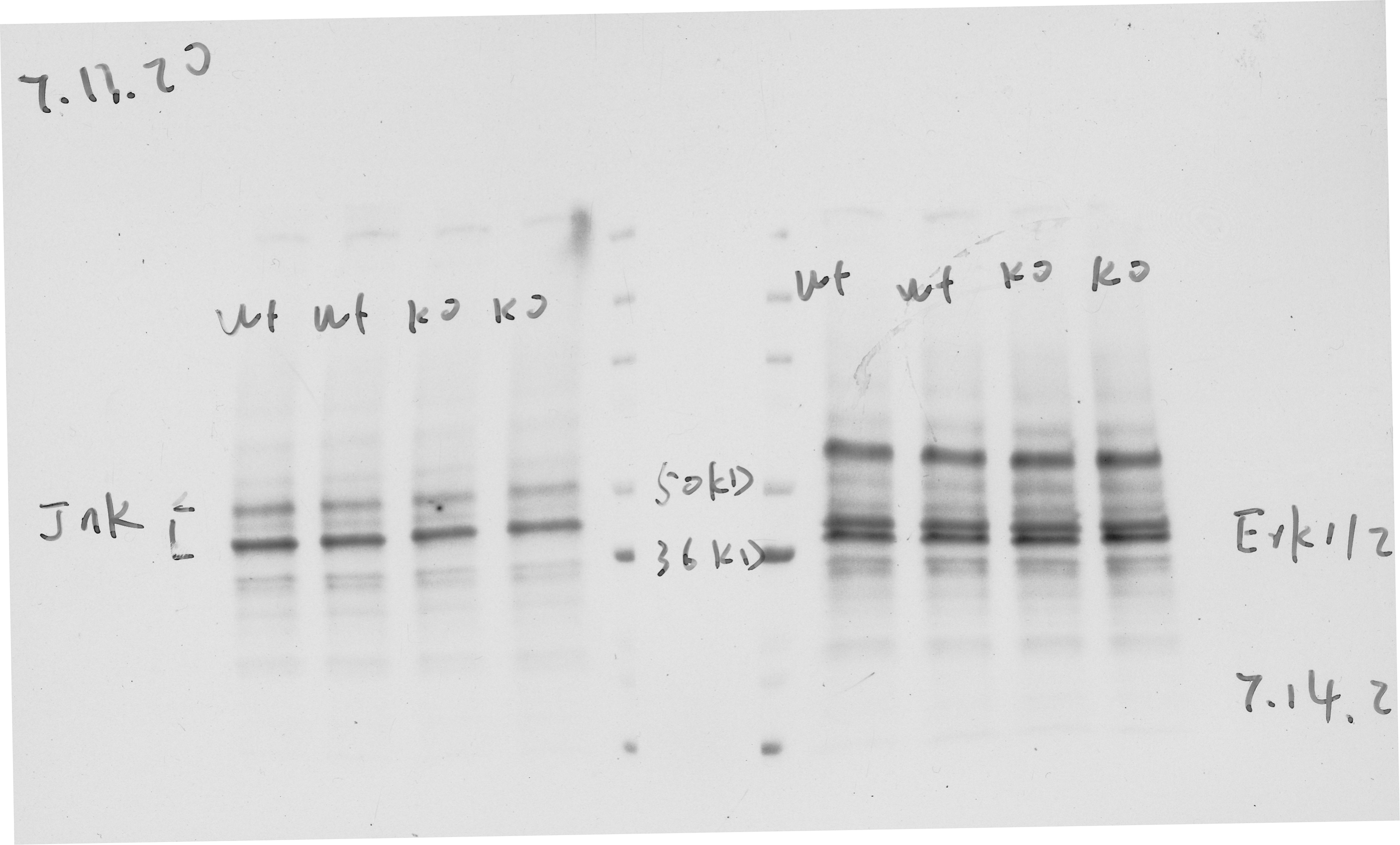

Supplement: Figure 4—source data 2. [file elife-91876-fig4-data2.zip › Figure 4-source data 2.tif]

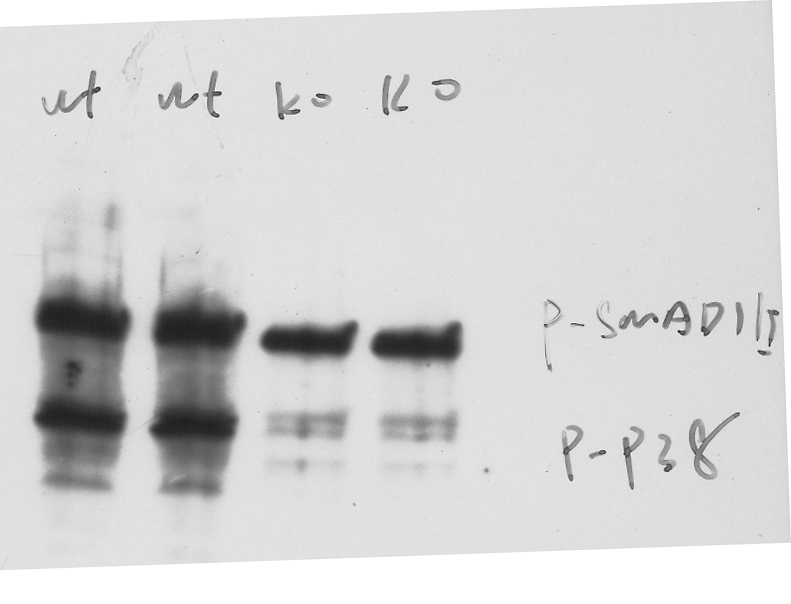

Supplement: Figure 4—source data 3. [file elife-91876-fig4-data3.zip › Figure 4-source data 3.tif]

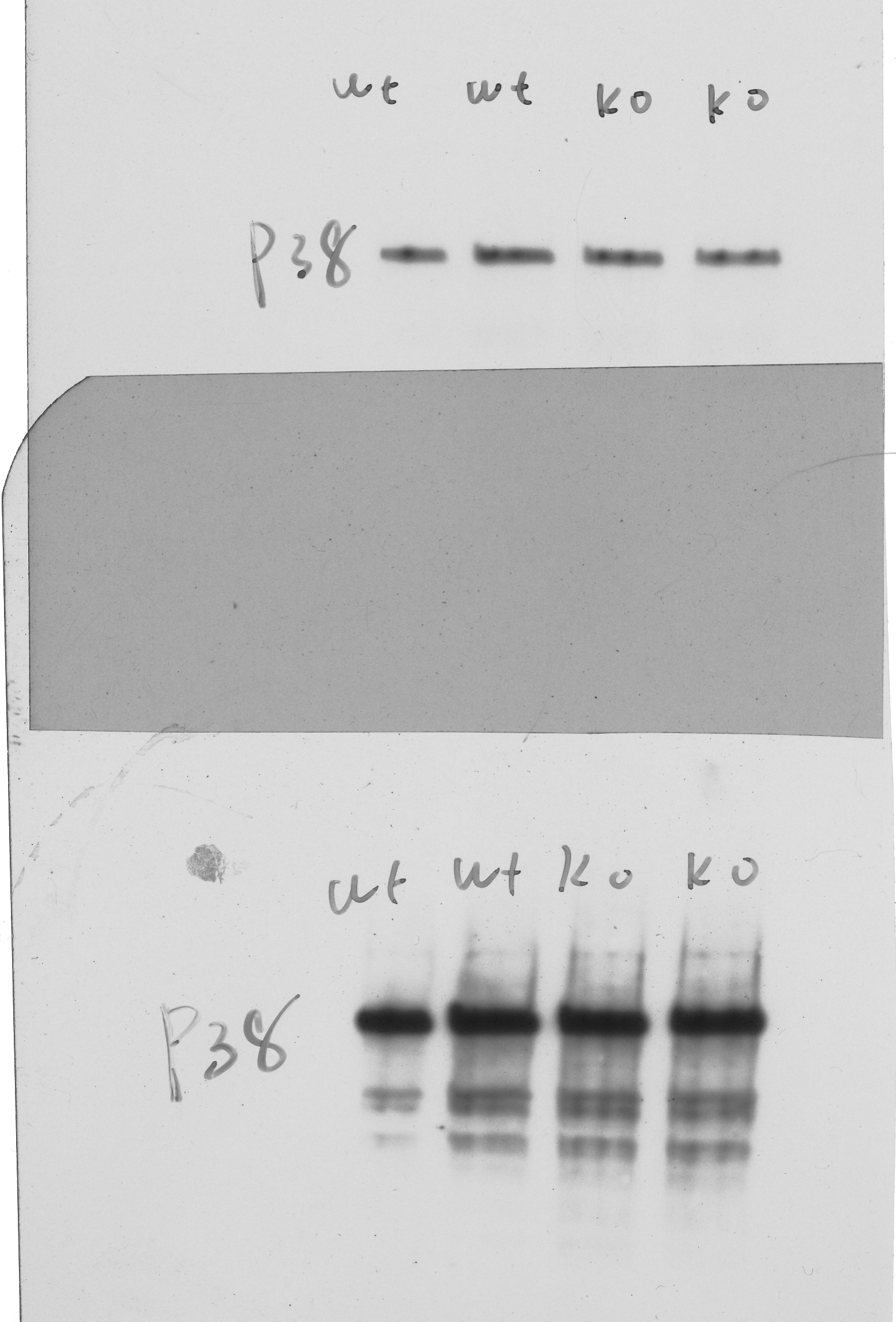

Supplement: Figure 4—source data 4. [file elife-91876-fig4-data4.zip › Figure 4-source data 4.tif]

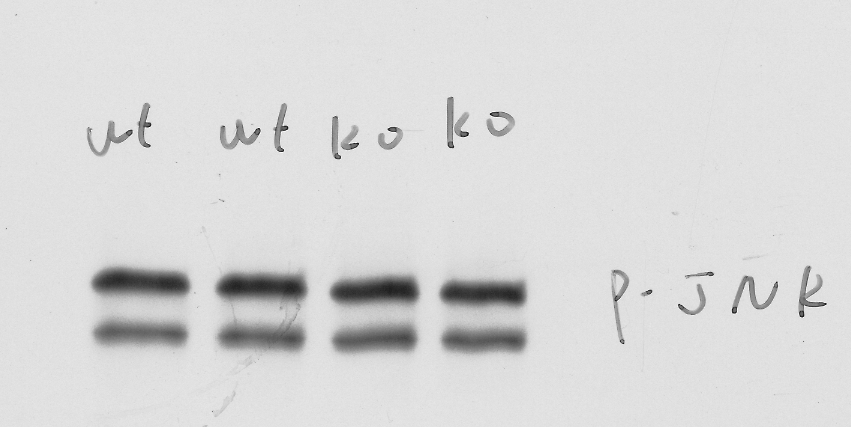

Supplement: Figure 4—source data 5. [file elife-91876-fig4-data5.zip › Figure 4-source data 5.tif]

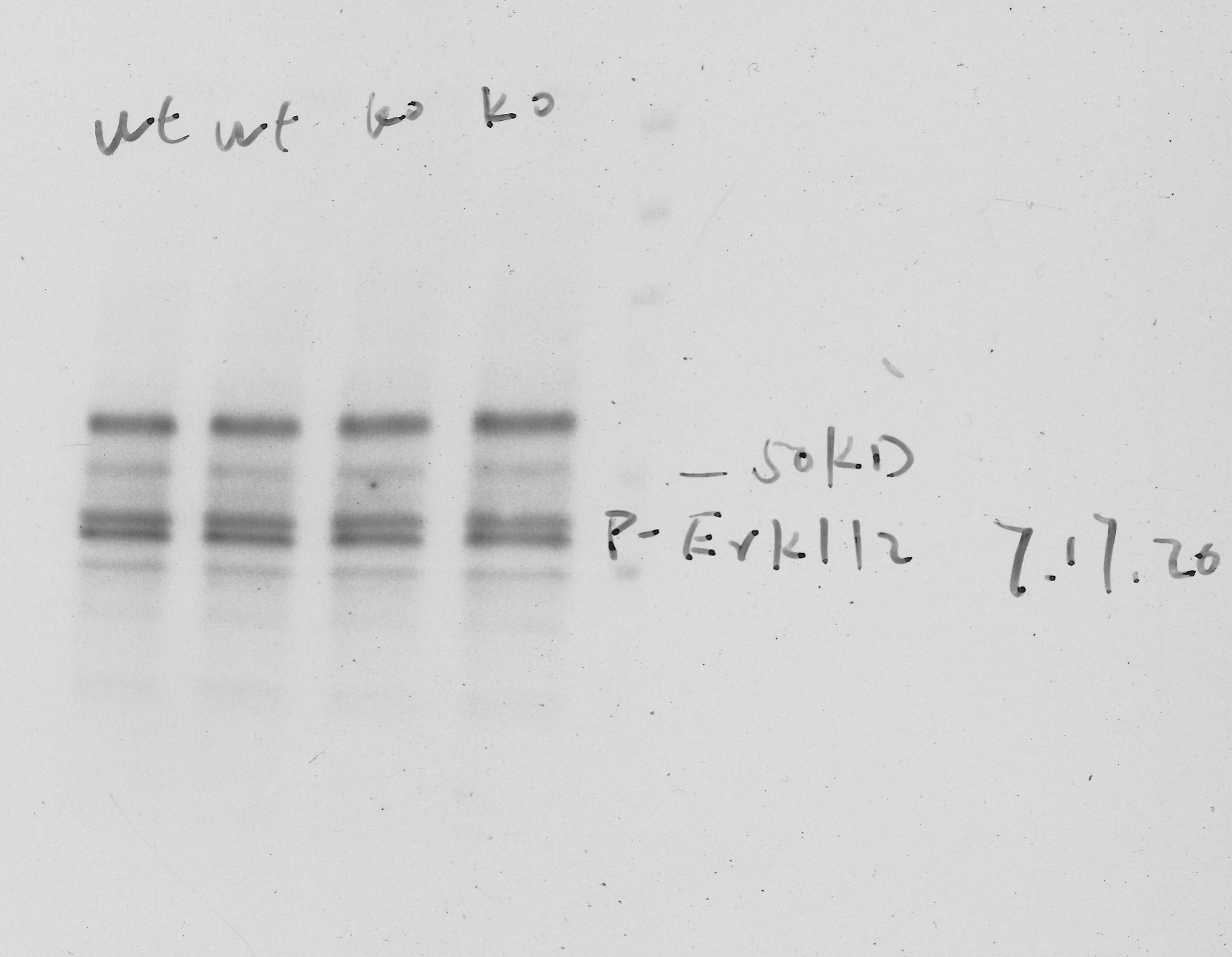

Supplement: Figure 4—source data 6. [file elife-91876-fig4-data6.zip › Figure 4-source data 6.tif]

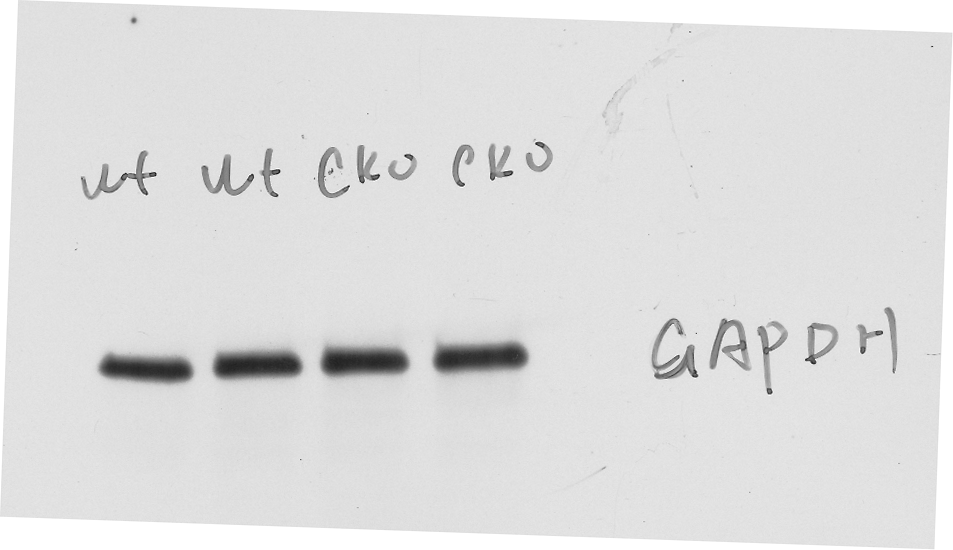

Supplement: Figure 4—source data 7. [file elife-91876-fig4-data7.zip › Figure 4-source data 7.tif]

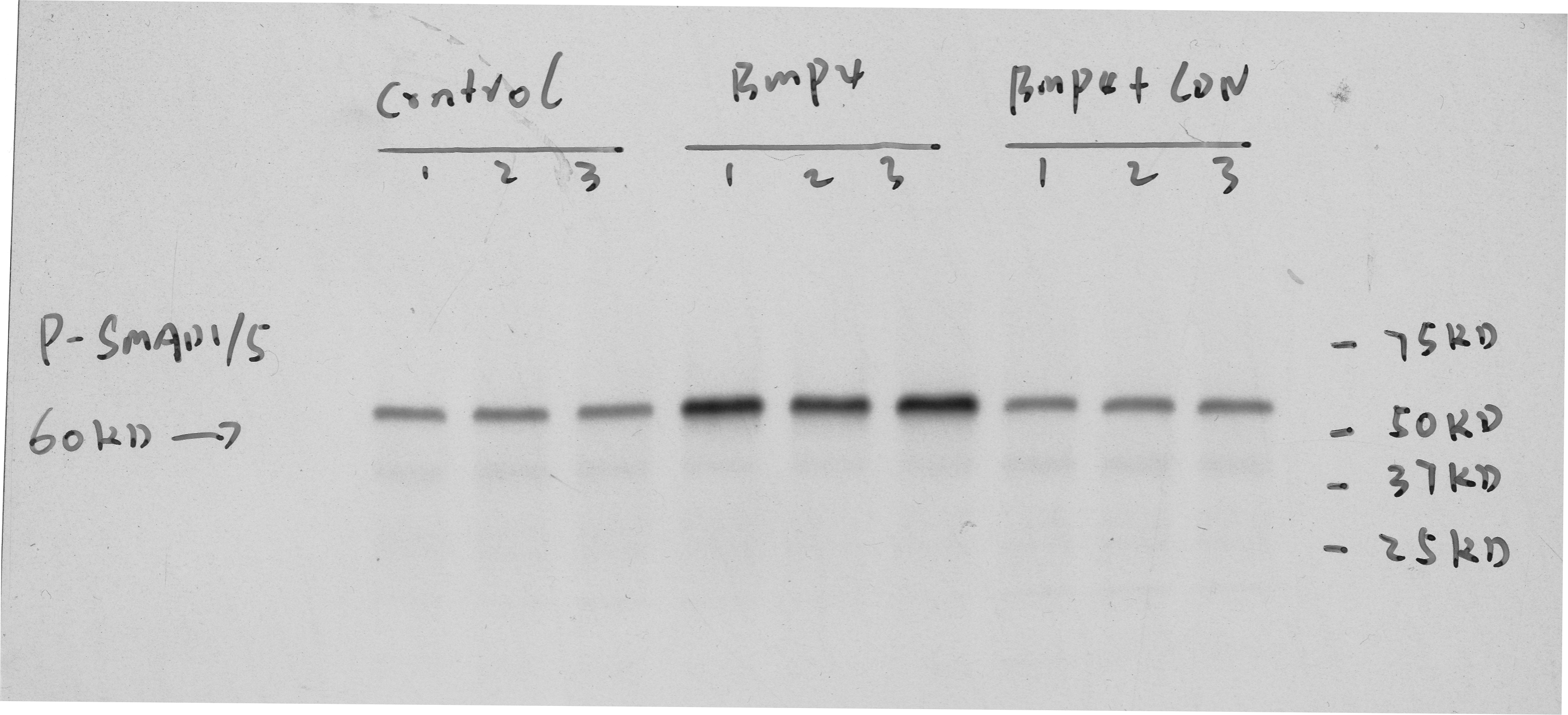

Supplement: Figure 4—source data 9. [file elife-91876-fig4-data9.zip › Figure 4-source data 9.tif]

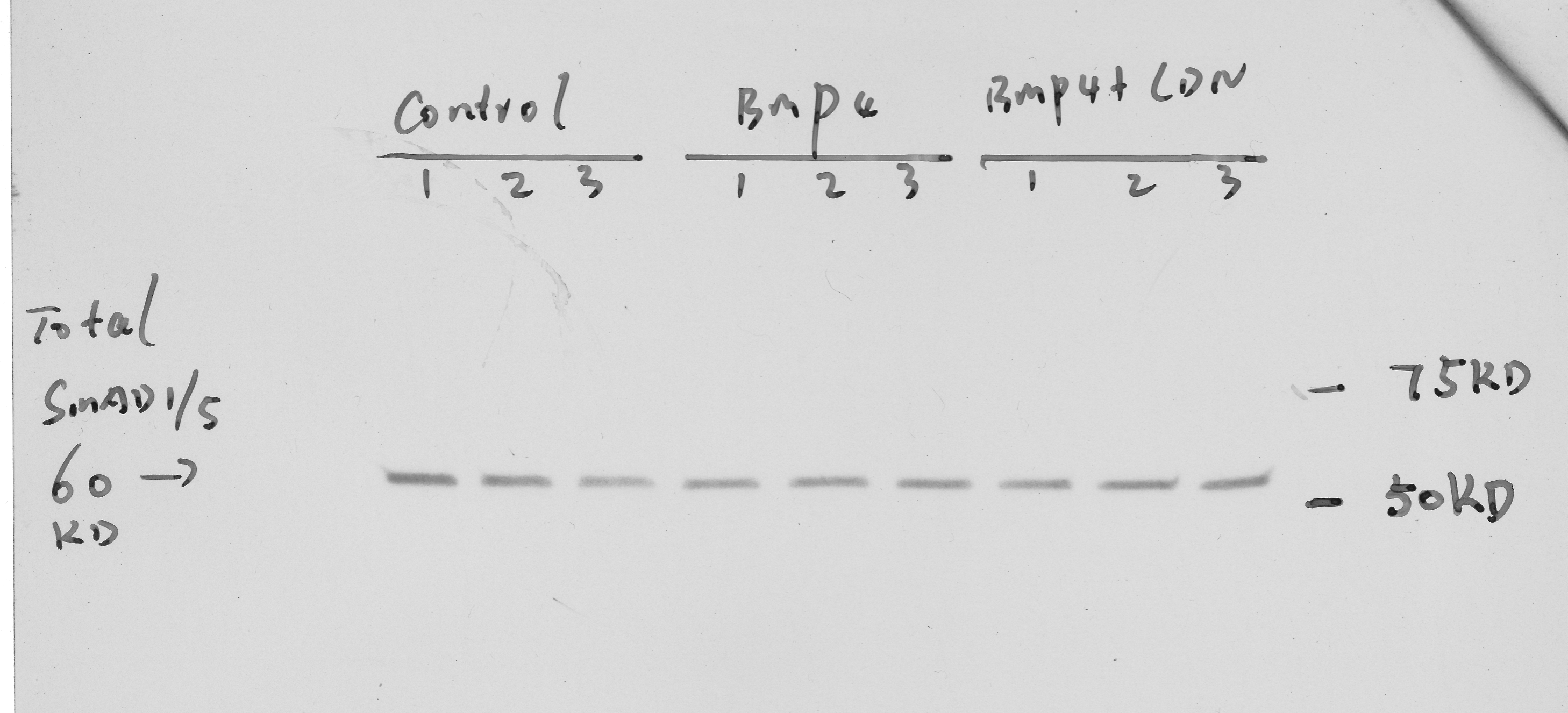

Supplement: Figure 4—source data 10. [file elife-91876-fig4-data10.zip › Figure 4-source data 10.tif]

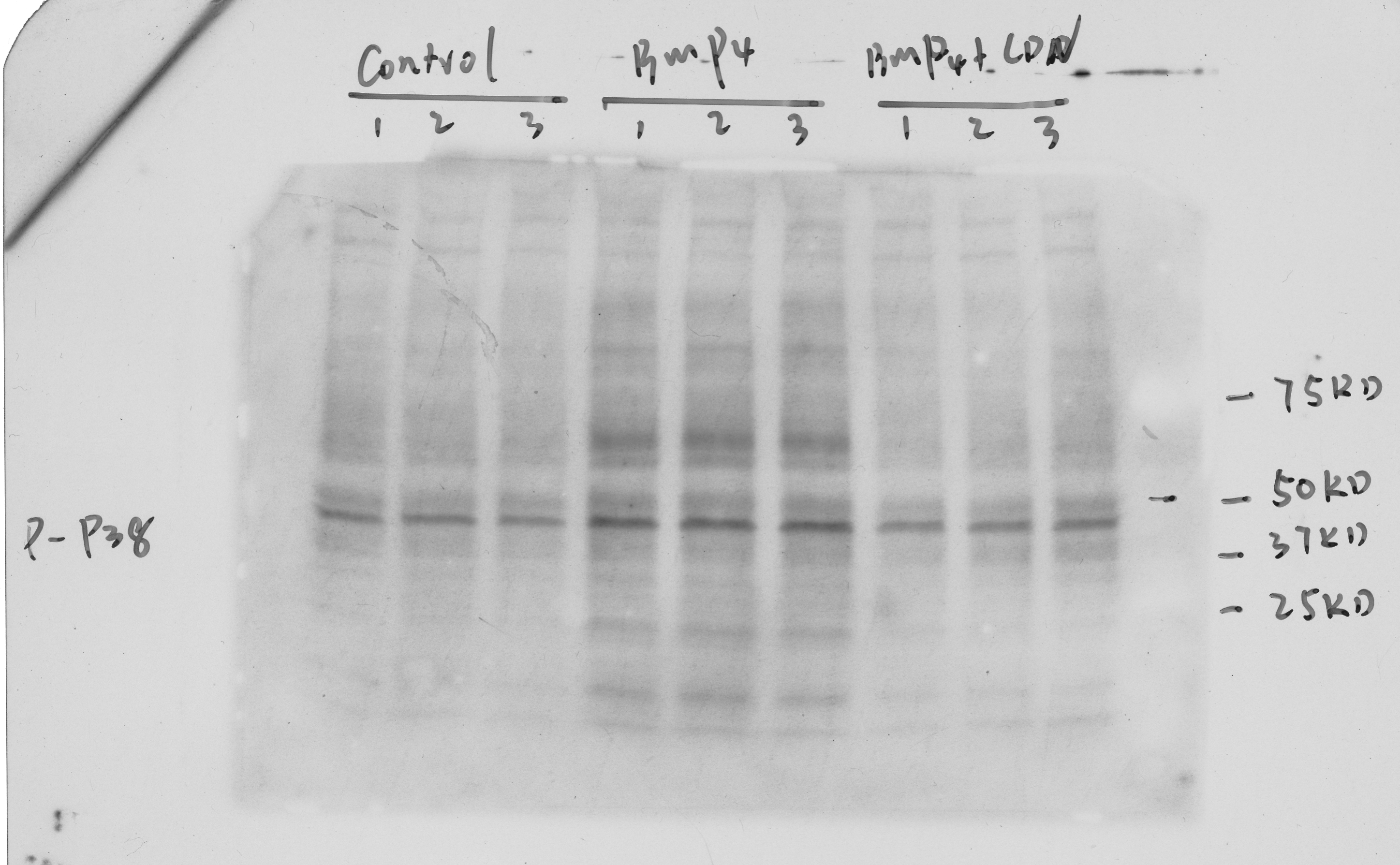

Supplement: Figure 4—source data 11. [file elife-91876-fig4-data11.zip › Figure 4-source data 11.tif]

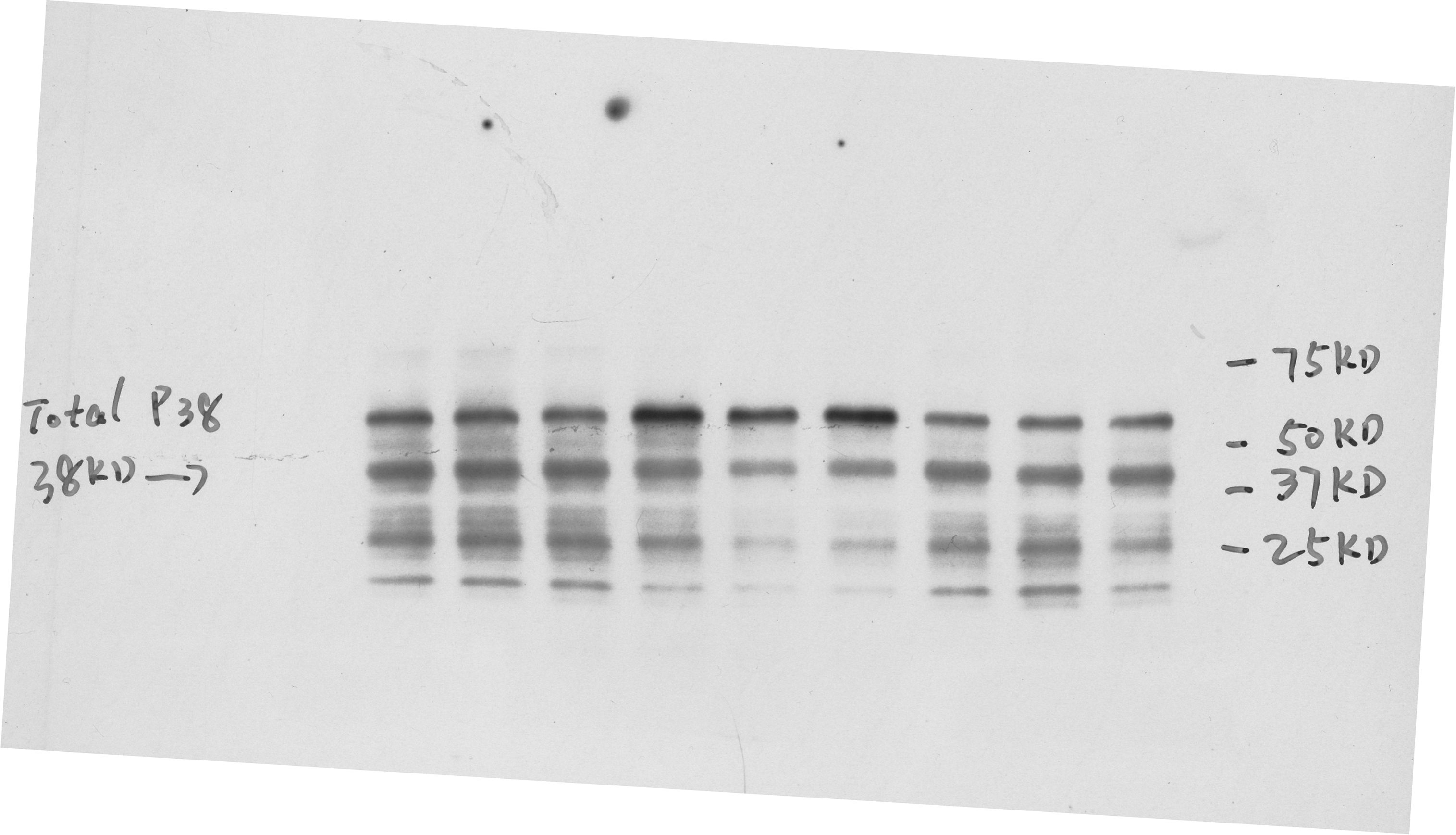

Supplement: Figure 4—source data 12. [file elife-91876-fig4-data12.zip › Figure 4-source data 12.tif]

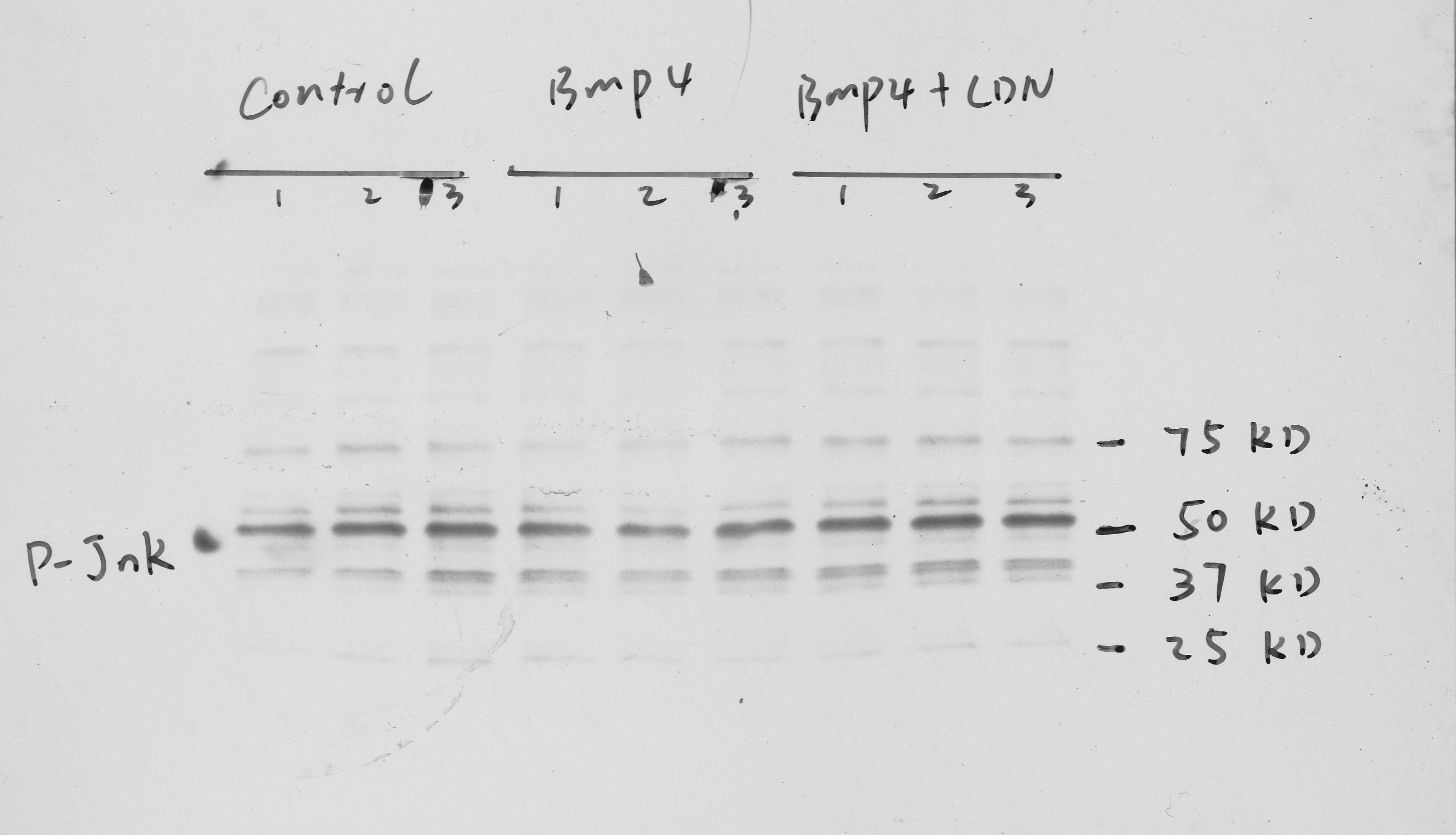

Supplement: Figure 4—source data 13. [file elife-91876-fig4-data13.zip › Figure 4-source data 13.tif]

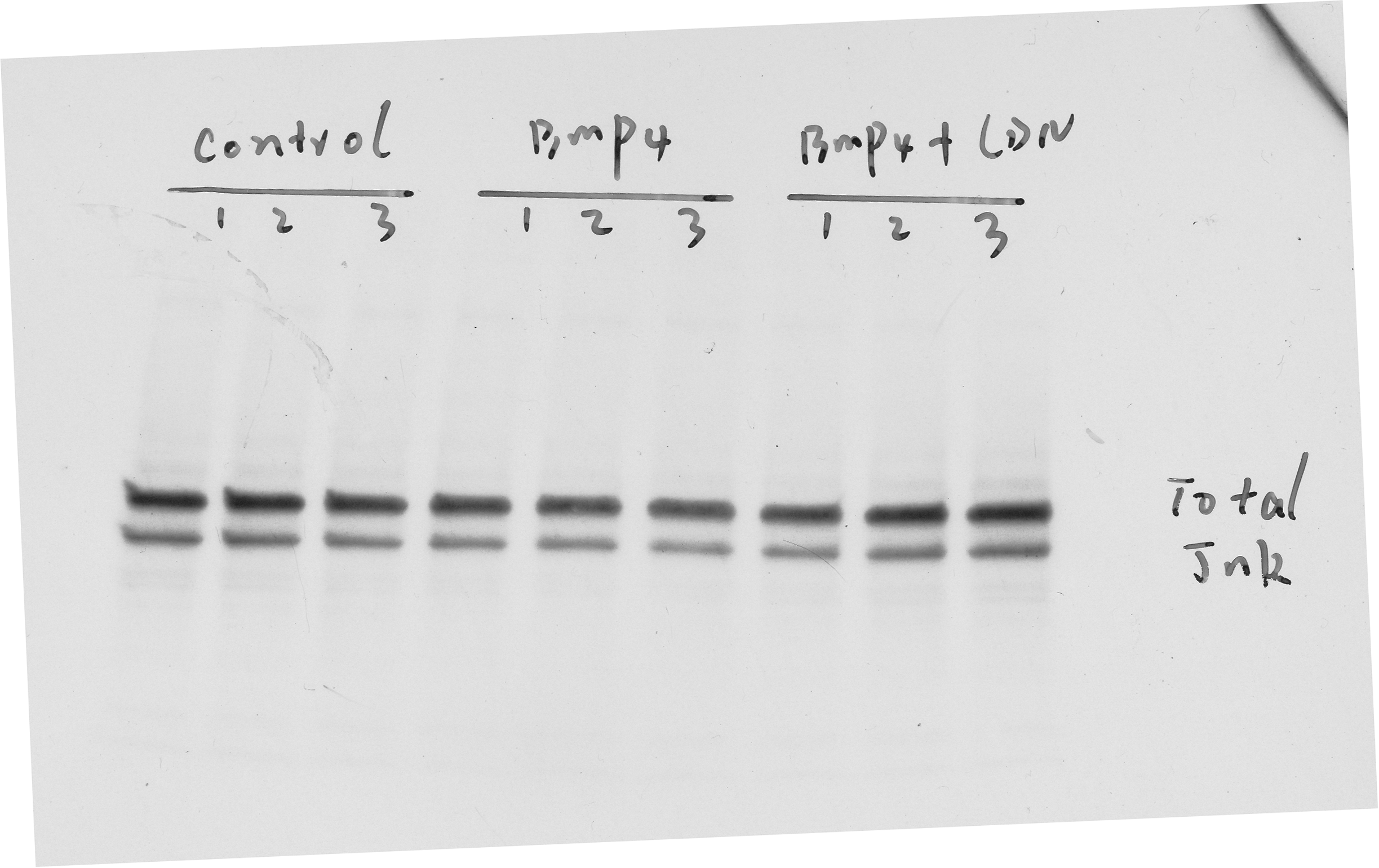

Supplement: Figure 4—source data 14. [file elife-91876-fig4-data14.zip › Figure 4-source data 14.tif]

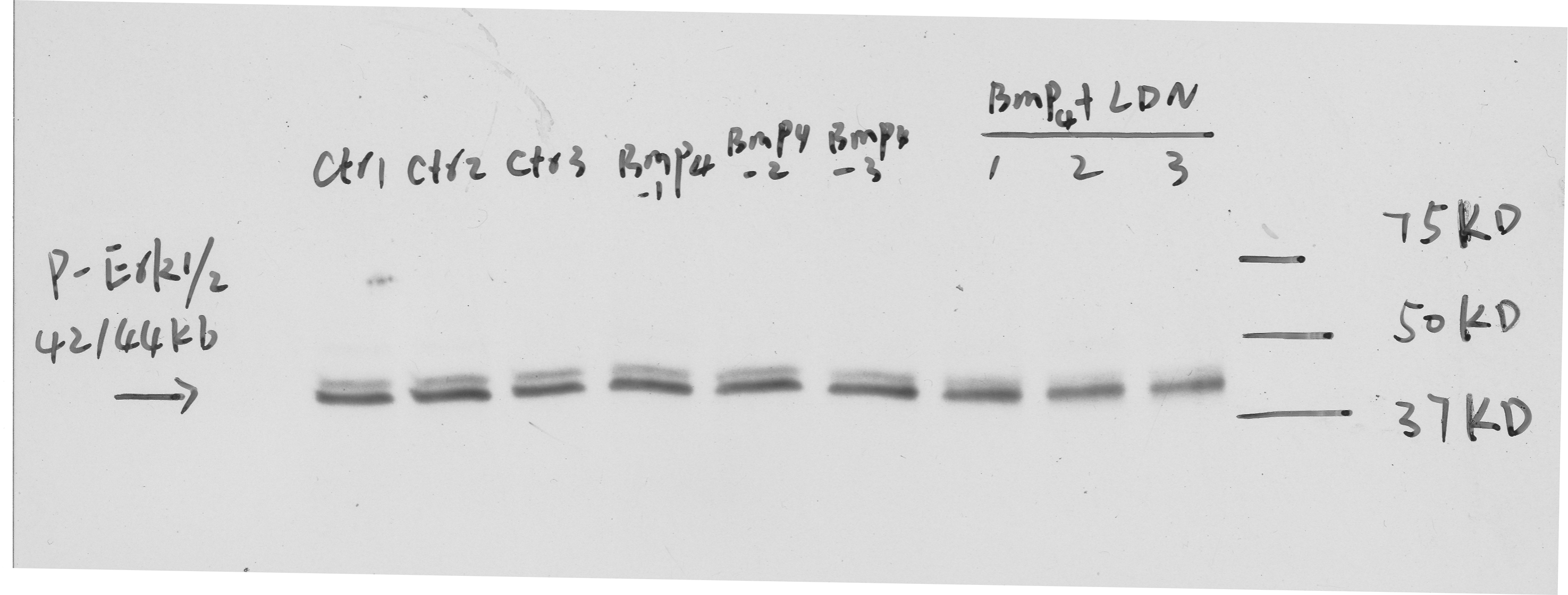

Supplement: Figure 4—source data 15. [file elife-91876-fig4-data15.zip › Figure 4-source data 15.tif]

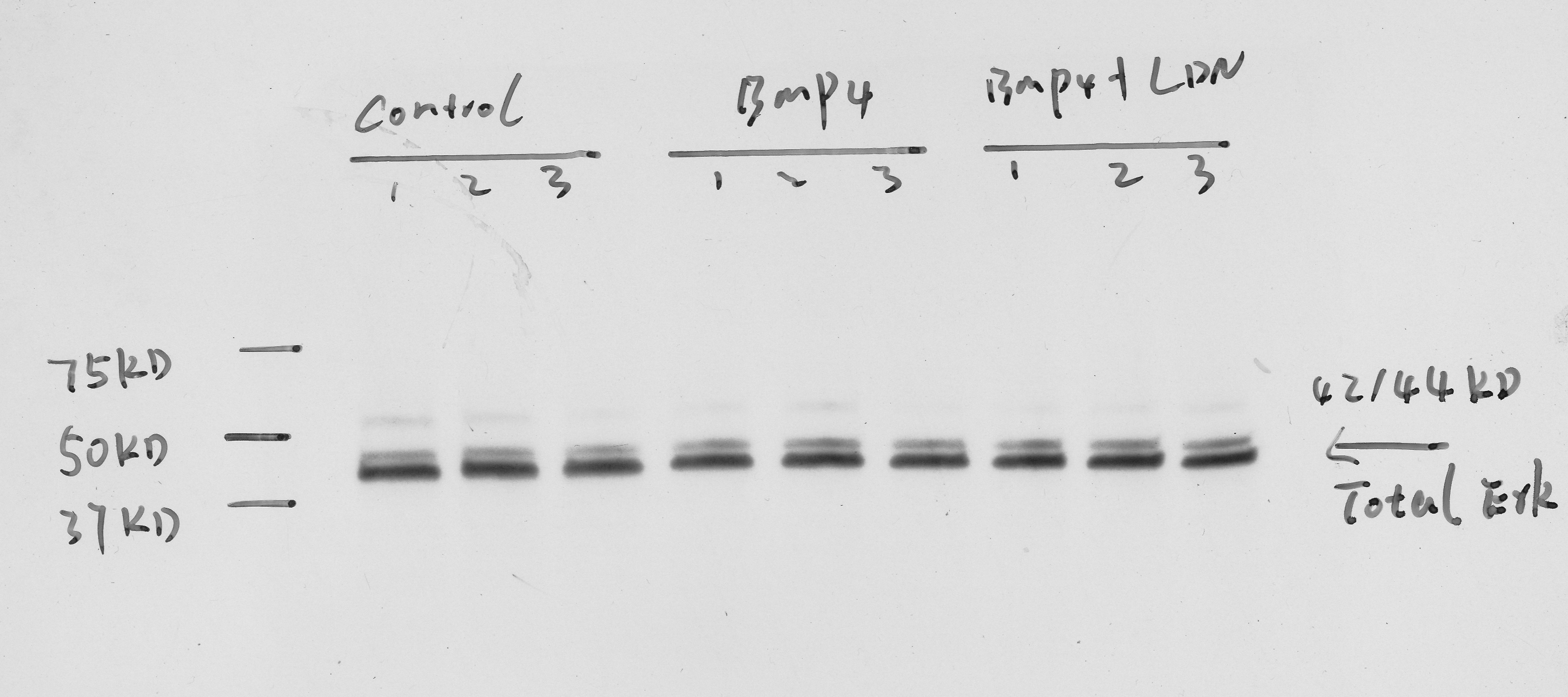

Supplement: Figure 4—source data 16. [file elife-91876-fig4-data16.zip › Figure 4-source data 16.tif]

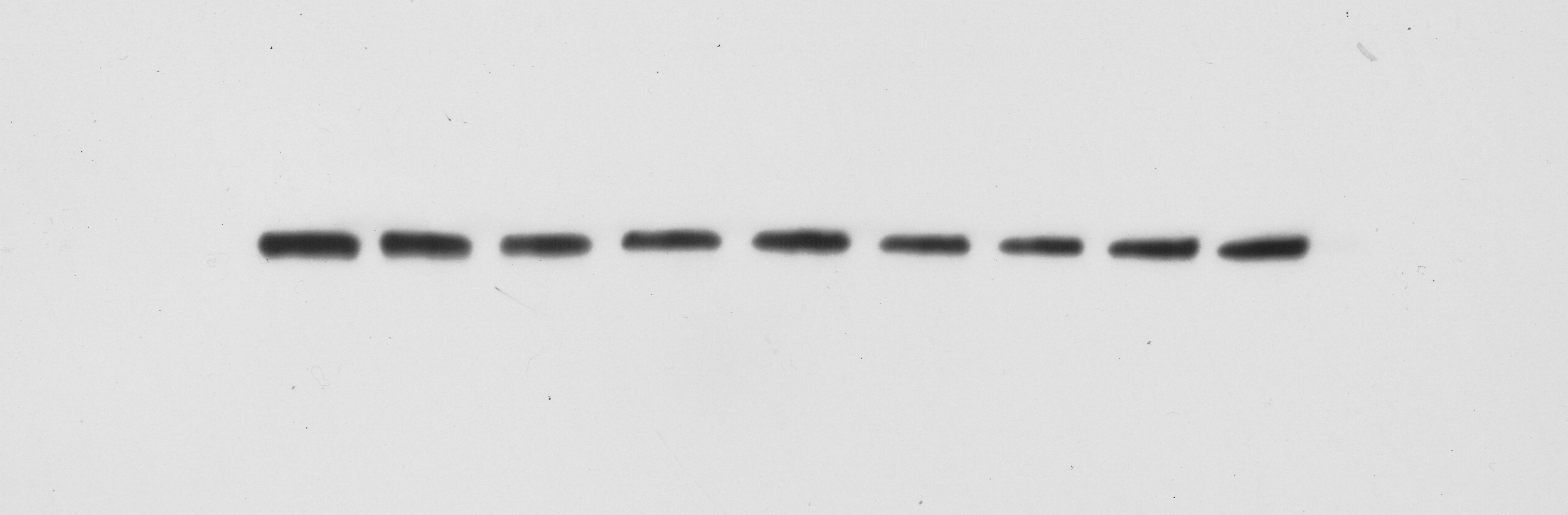

Supplement: Figure 4—source data 17. [file elife-91876-fig4-data17.zip › Figure 4-source data 17.tif]

E

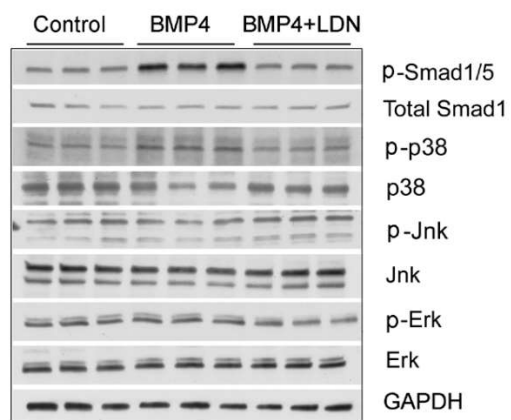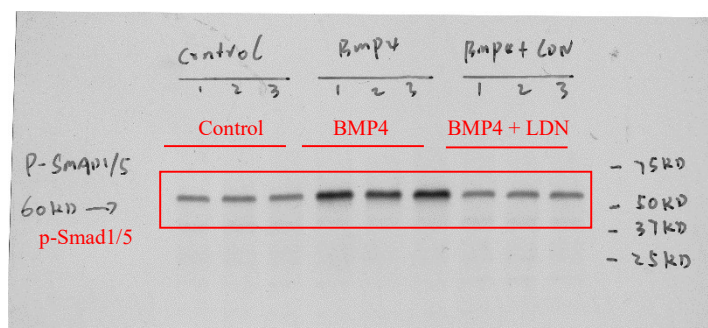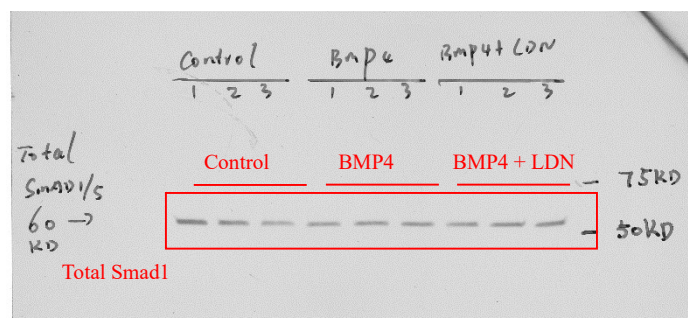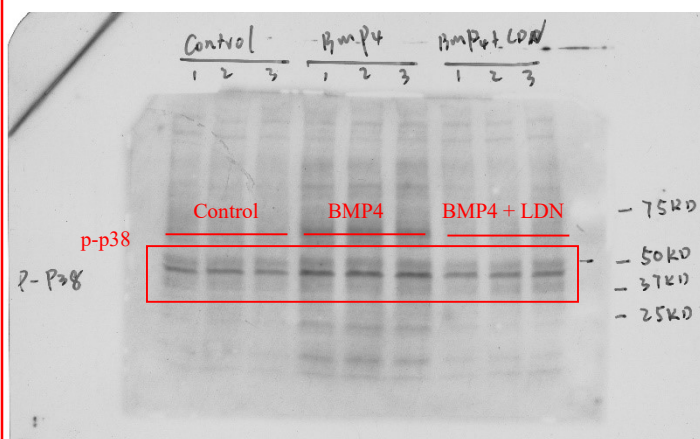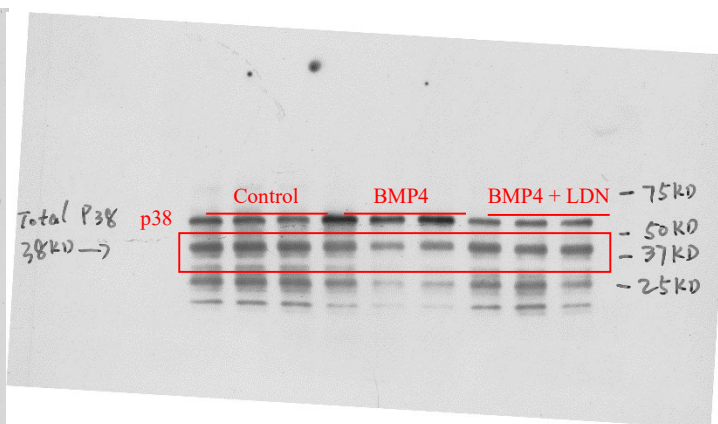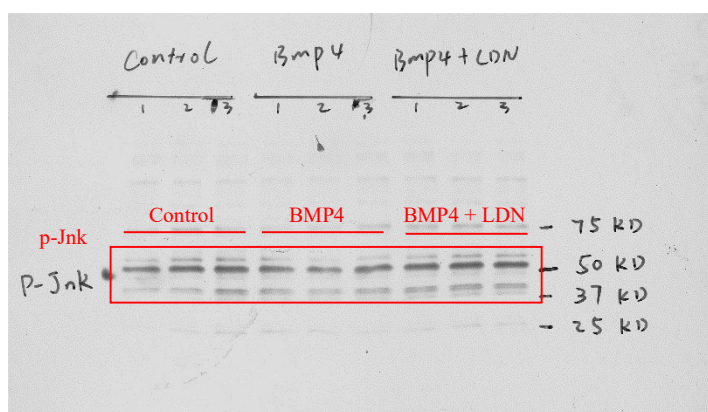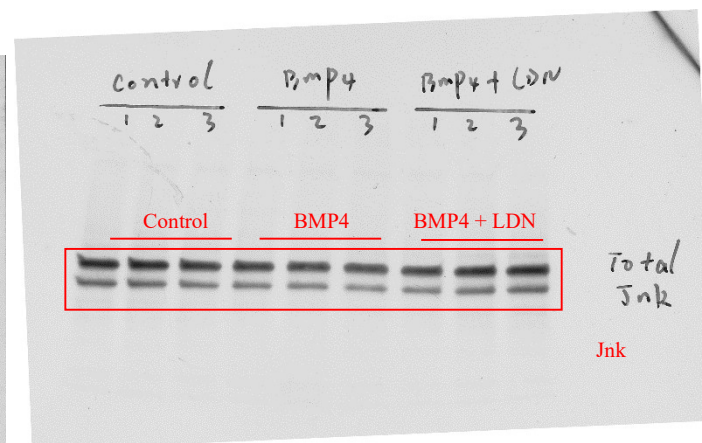

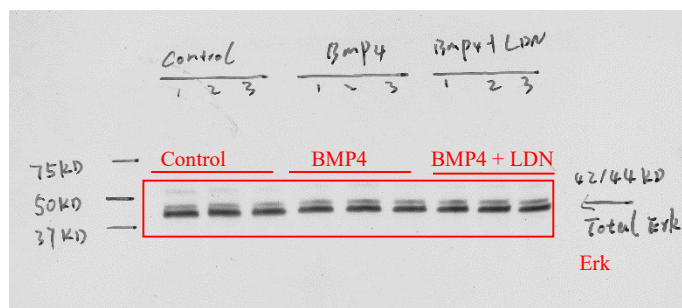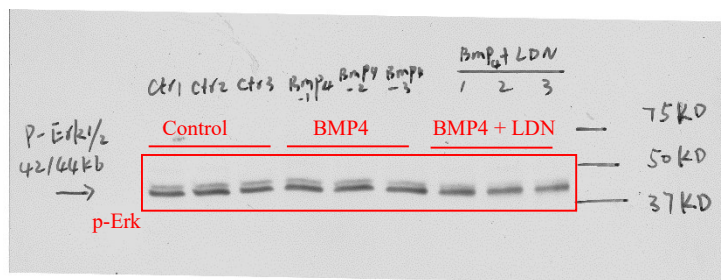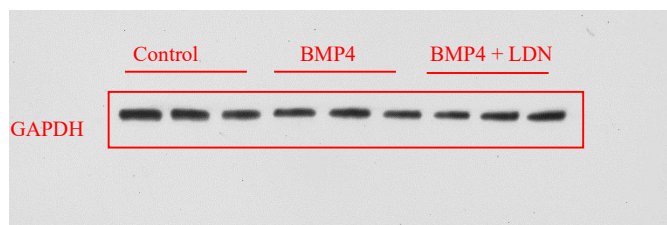

Figure 4

Supplement: Figure 4—source data 18. [file elife-91876-fig4-data18.zip › Figure 4-source data 18.pdf]

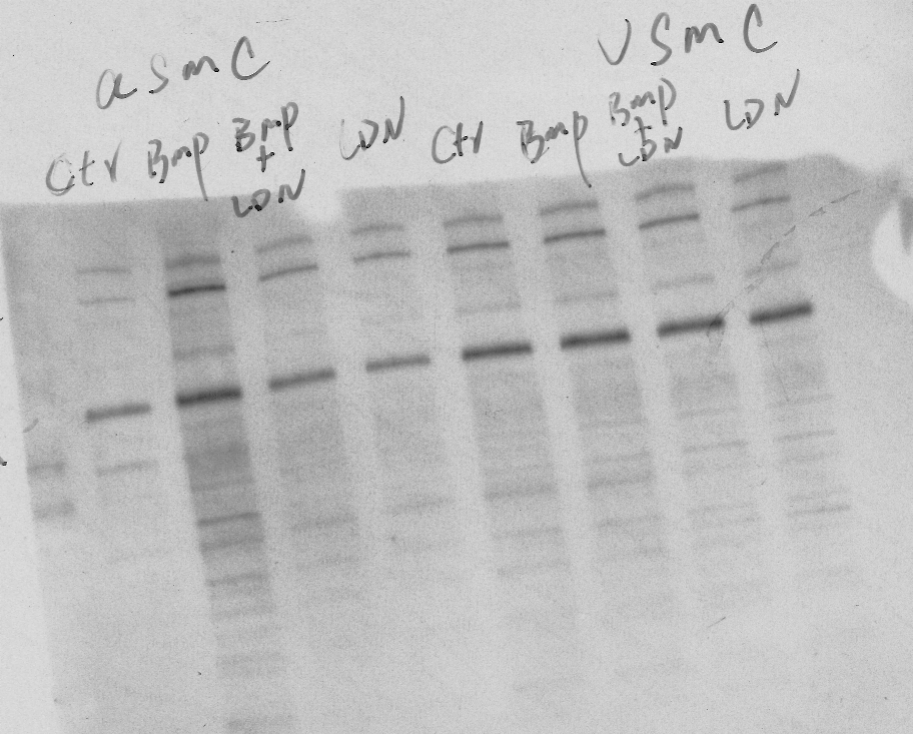

Supplement: Figure 5—source data 1. [file elife-91876-fig5-data1.zip › Figure 5-source data 1.tif]

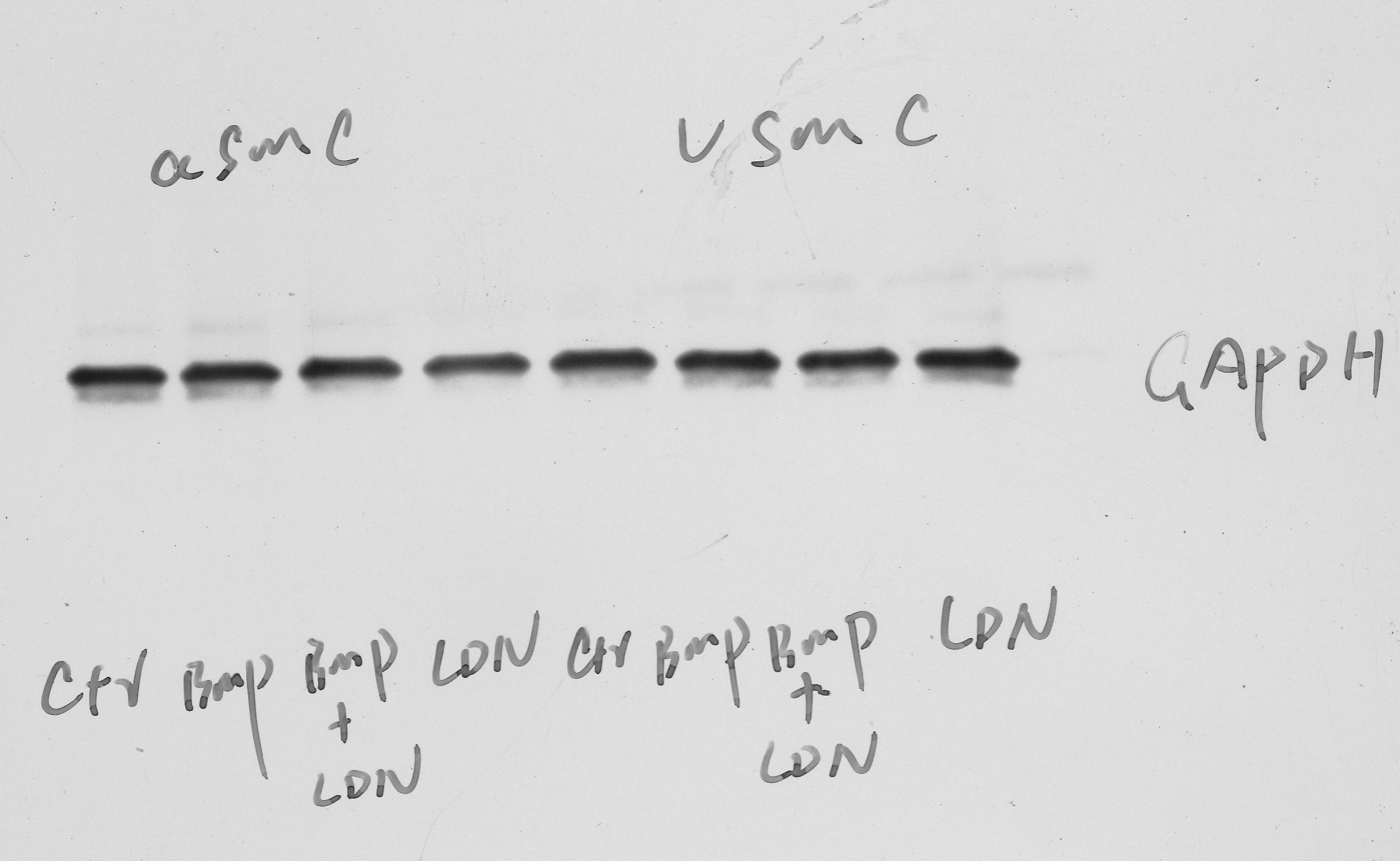

Supplement: Figure 5—source data 2. [file elife-91876-fig5-data2.zip › Figure 5-source data 2.tif]

C

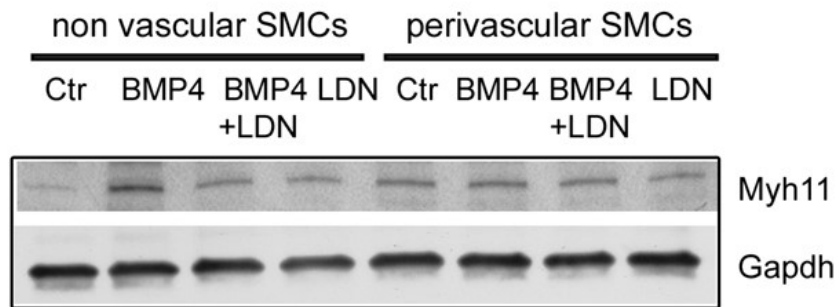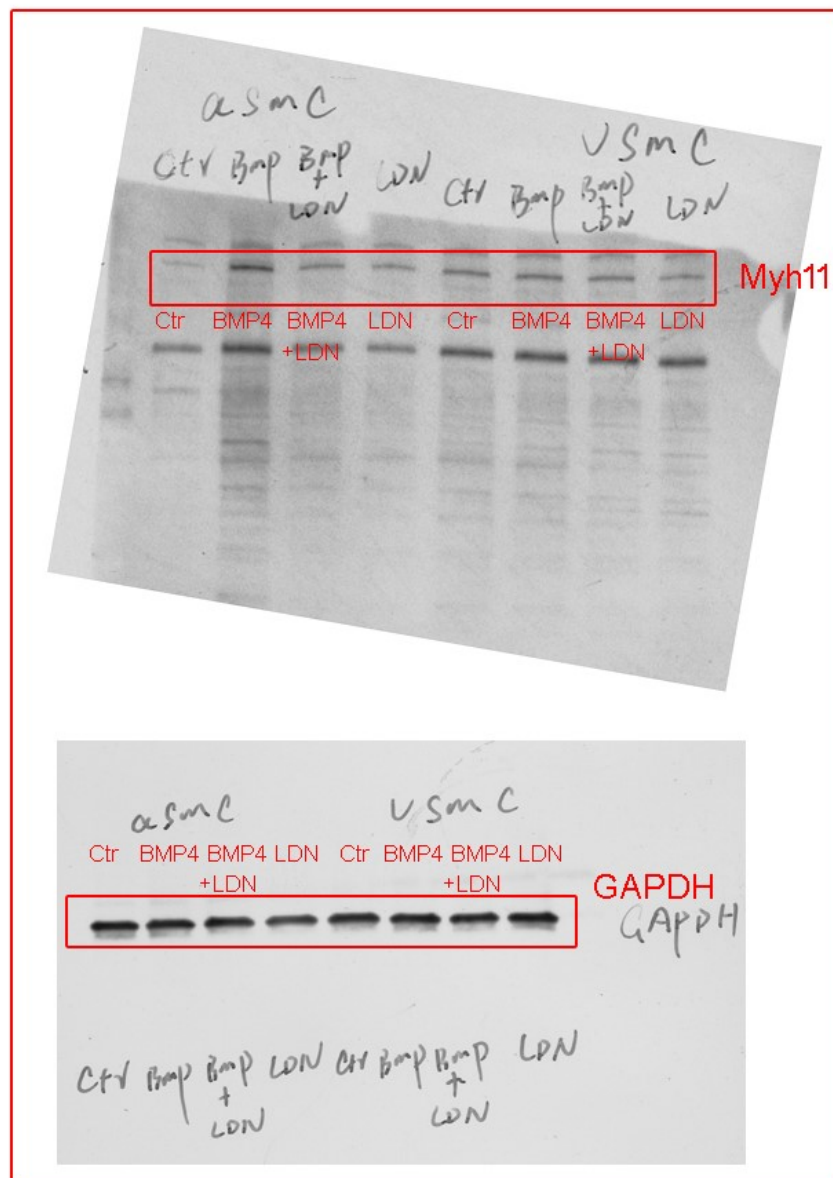

Figure 5

Supplement: Figure 5—source data 3. [file elife-91876-fig5-data3.zip › Figure 5-source data 3.pdf]

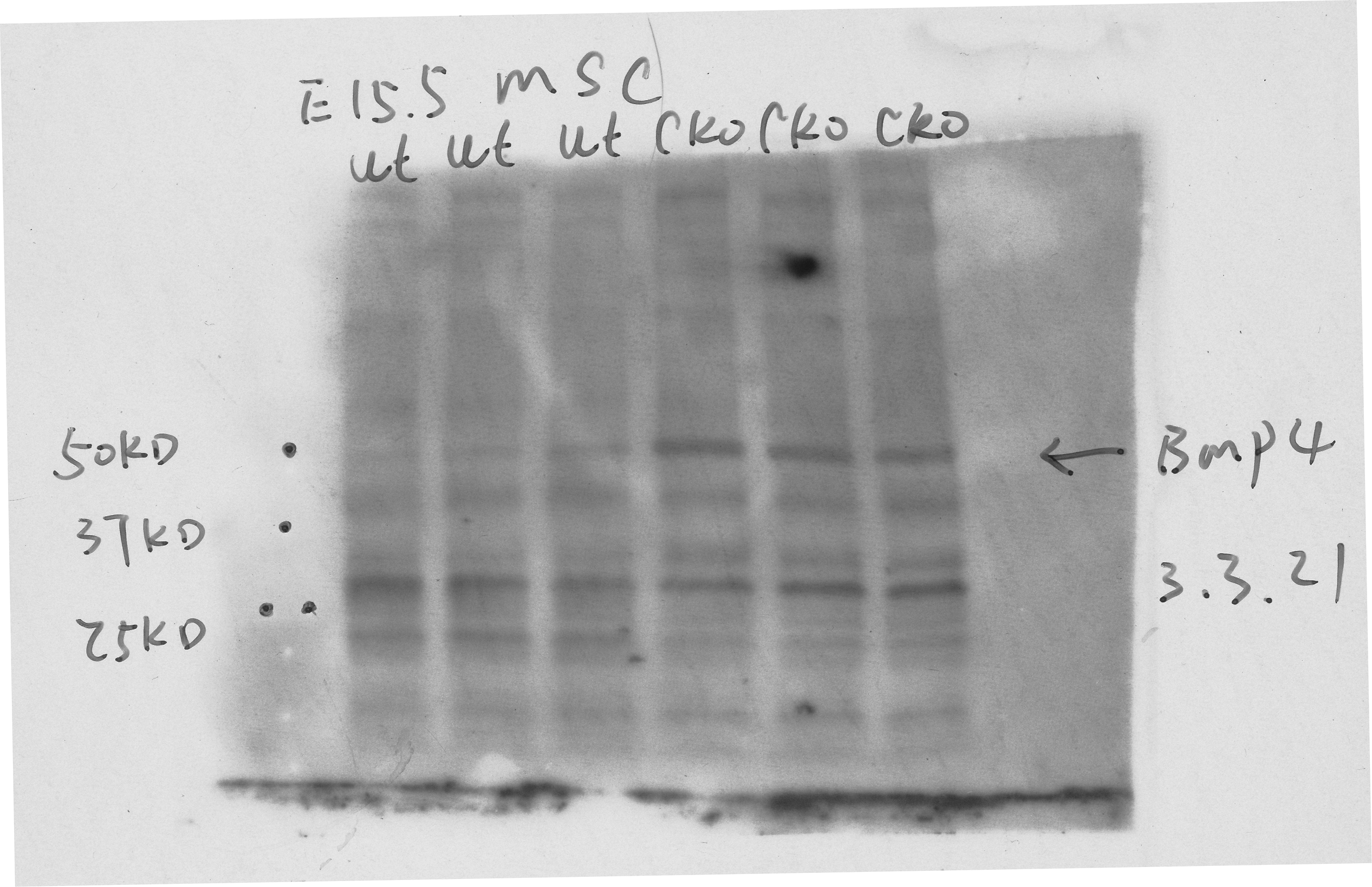

Supplement: Figure 7—source data 1. [file elife-91876-fig7-data1.zip › Figure 7-source data 1.tif]

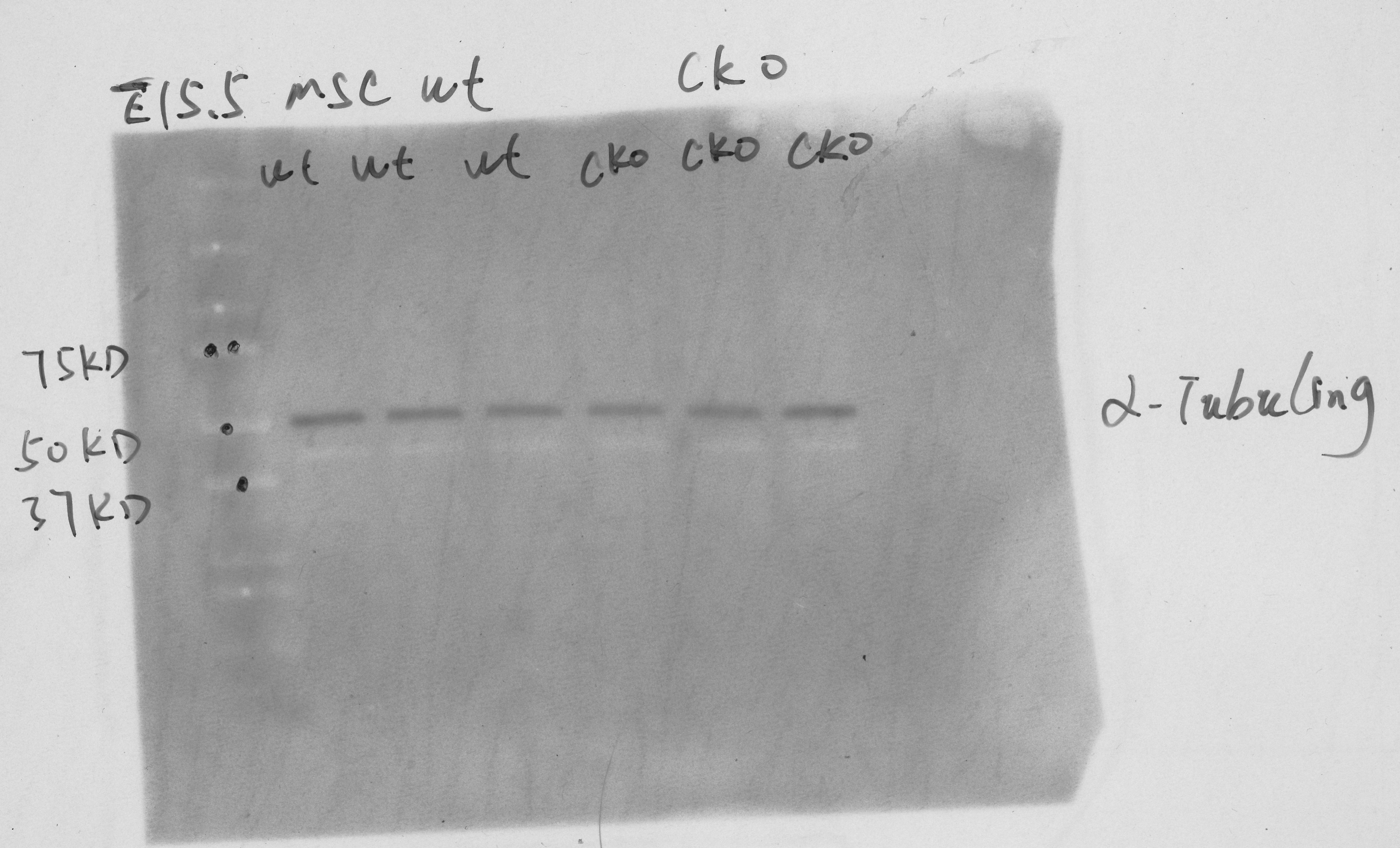

Supplement: Figure 7—source data 2. [file elife-91876-fig7-data2.zip › Figure 7-source data 2.tif]

E

E15.5 MPCs

WT

*Bmpr1a* CKO

Bmp4

$\alpha$ -Tubulin

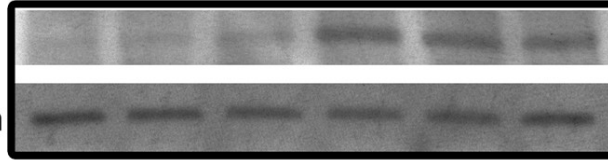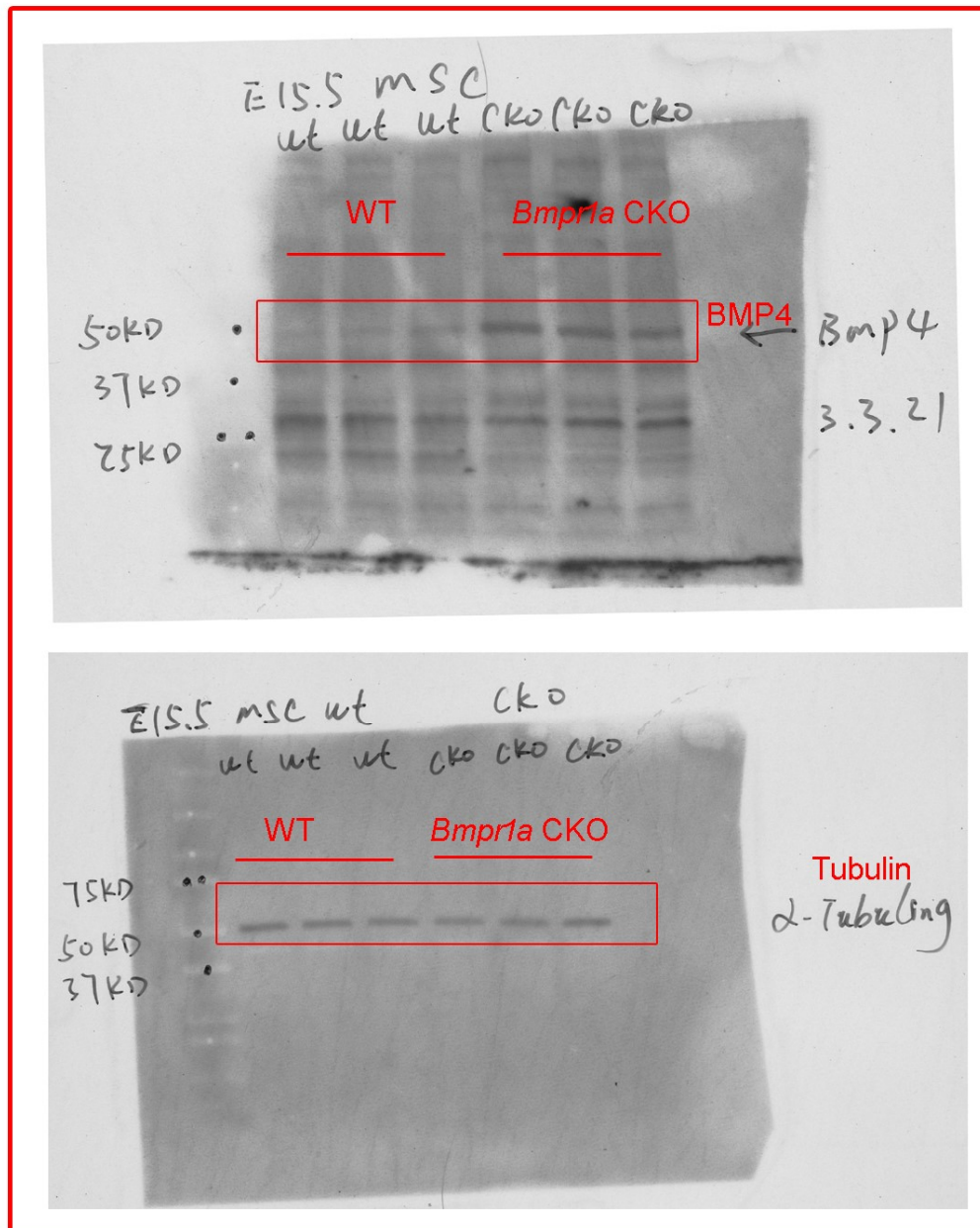

Figure 7

Supplement: Figure 7—source data 3. [file elife-91876-fig7-data3.zip › Figure 7-source data 3.pdf]
