# Supplementary material for: Defective mesenchymal Bmpr1a-mediated BMP signaling causes congenital pulmonary cysts
Source: eLife. 2024 Jun 10;12:RP91876. doi: 10.7554/eLife.91876 (PMC11164533; doi:10.7554/eLife.91876)
Supplement: Figure 4—source data 8. [file elife-91876-fig4-data8.zip › Figure 4-source data 8.pdf]

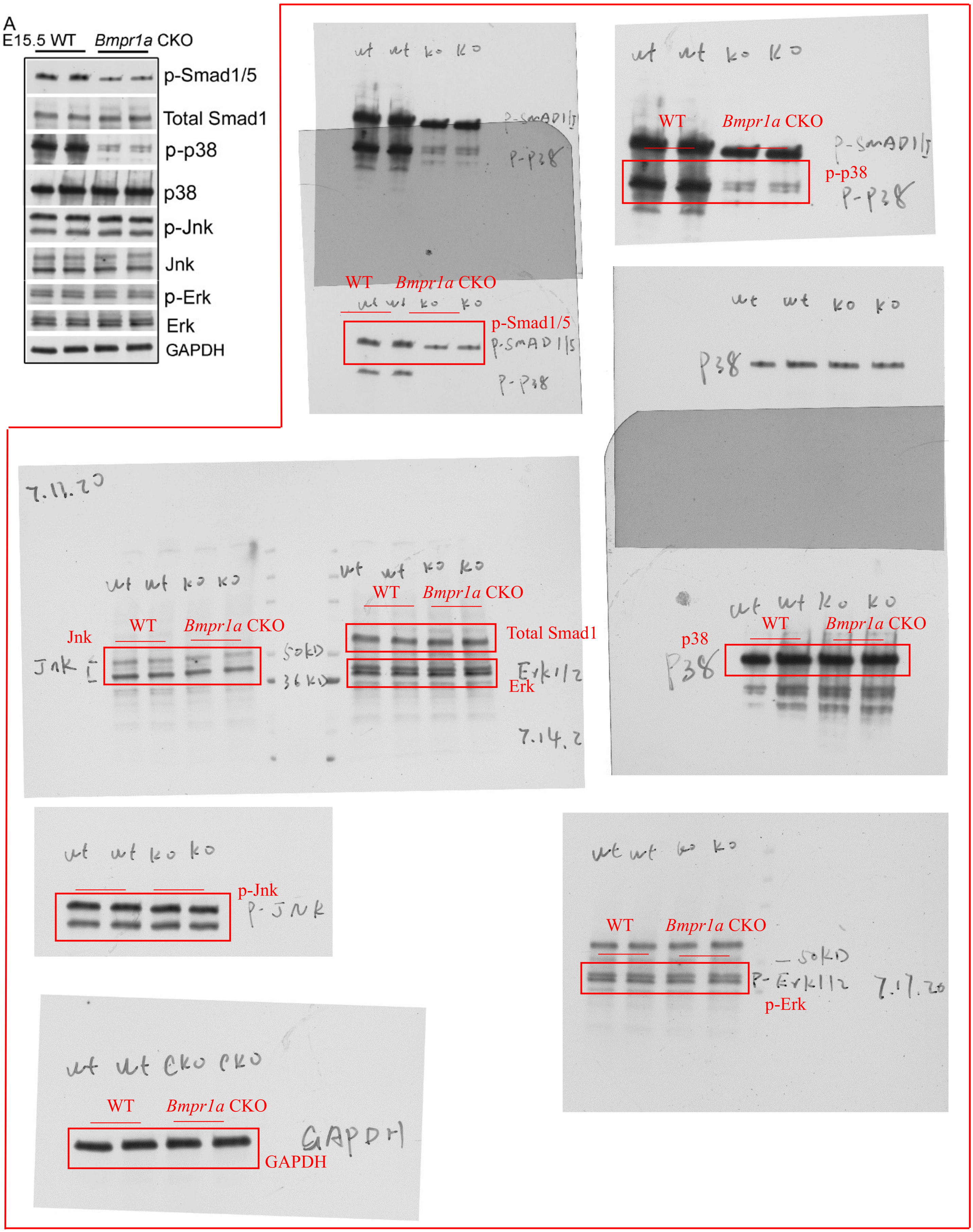
A
E15.5 WT
Bmpr1a CKO
p-Smad1/5
Total Smad1
p-p38
p38
p-Jnk
Jnk
p-Erk
Erk
GAPDH
WT
Bmpr1a CKO
p-Smad1/5
p-p38
p38
Jnk
Total Smad1
Erk
p-Jnk
p-Erk
GAPDH

Figure 4
